# Supplementary material for: Dynamic Tracking of Native Polyclonal Hematopoiesis in Adult Mice
Source: bioRxiv. 2024 Apr 3:2024.04.02.587737. Preprint. [Version 1] doi: 10.1101/2024.04.02.587737 (PMC11014561; doi:10.1101/2024.04.02.587737)
Supplement: Supplementary file 1 [file NIHPP2024.04.02.587737v1-supplement-1.pdf]

bioRxiv preprint doi: https://doi.org/10.1101/2024.04.02.587737; this version posted April 3, 2024. The copyright holder for this preprint (which was not certified by peer review) is the author/funder, who has granted bioRxiv a license to display the preprint in perpetuity. It is made available under aCC-BY 4.0 International license.

<u>Supplementary Figure Legend</u>

Figure S1: Establishing the correlation between variance of FP and precursor numbers.

(A) The correlation between precursor numbers and CVs of individual FP or all CVs (original correlation formula), replotted from Ganuza et al., 2017.

(B) FP_{mean}% in a previously published dataset, replotted from Ganuza et al., 2017.

(C) The correlation between precursor numbers and standard deviation (σ) of individual FP, replotted from Ganuza et al., 2017.

(D) The BFP% in HL-60 mix during two weeks of growth.

(E) The effect of outliers on precursor number estimation. Left, BFP% in samples with/without outliers. Quartiles by Tukey method was shown. The outlier is highlighted by circle. Middle, standard deviation of BFP% with/without outliers. Right, estimated precursor numbers with/without outliers.

(F) The changes of standard deviation when recorded events varied. “&” denotes the potential overestimated standard deviation due to low flow recorded events.

(G) The variance of estimated precursor numbers when varying sample sizes were used.

(H) The relative error of estimation as sample sizes increased. Each dot represents data from one seeded number. Error bars represent mean ± SD.

Figure S2: The induction specificity of HSC-Scl-$CreER^T$

(A) Experiment schematic to examine the induction specificity of HSC-Scl-$CreER^T$. N =10 for induced animals, N =3 for non-induced animals. Image was created with Biorender.

(B) Flow gating strategy for BM HSPC and MPs.

(C) Confetti labeling in BM HSPCs. Error bars represent mean ± SD.

(D) Flow gating strategy for BM lineage cells.

(E) Confetti labeling in BM lineage cells. Error bars represent mean ± SD.

(F) Flow gating strategy to examine spleen cells.

(G) Confetti labeling in spleen cells. Error bars represent mean ± SD.

See Methods and Table S1 for the detailed antibody, fluorophore and marker combination used for each cell type.

Figure S3: Analysis of HSC-Scl-$CreER^T$ background Cre activity

(A) Representative flow plot showing Confetti expression in PB T cells in 48-50-week-old HSC-Scl-$CreER^T$/Confetti animals compared to an animal induced with one-day tamoxifen treatment.

(B) Quantification of RFP and YFP% in PB T cells. N =8 for non-induced 48-50-week-old animals.

bioRxiv preprint doi: https://doi.org/10.1101/2024.04.02.587737; this version posted April 3, 2024. The copyright holder for this preprint (which was not certified by peer review) is the author/funder, who has granted bioRxiv a license to display the preprint in perpetuity. It is made available under aCC-BY 4.0 International license.

(C) Representative flow plot for Confetti labeling in PB T cells post HSC-Scl-CreER^T-mediated induction.

(H) The level of CFP$^+$ and RFP$^+$ double positive cells in PB T cells of animals induced by HSC-Scl-CreER^T compared to Vav-Cre/Confetti animals analyzed at the same age. N =3 for Vav-Cre animals, N= 8 for HSC-Scl-CreER^T animals.

Figure S4: Variance of FP% inversely correlates with precursor numbers *in vivo*

(A) The correlation between expected numbers and the estimated numbers calculated from FP% of precursors following Poisson distribution. See Methods for simulation details.

(B) Flow gating strategy for PB B cell, T cell and myeloid cell. See Methods and Table S1 for the detailed antibody, fluorophore and marker combination used for each cell type.

(C) RFP_{Conf}% distribution in B cell and T cell.

(D) YFP_{Conf}% distribution in myeloid cell.

(E) CFP_{Conf}% distribution in myeloid cell.

(F) RFP_{PB}%, CFP_{PB}%, YFP_{PB}% and Confetti% distribution in myeloid cells.

(G) Linear correlation of PB precursor numbers calculated from RFP_{Conf}%, RFP_{PB}%, CFP_{PB}%, YFP_{PB}%, Confetti% or $RFP_{RFP+YFP}$%, Each dot represents a precursor number calculated from multiple mice.

For (C-F), each dot represents one animal. Recipient sample size = 7-14 mice per replicate, N =3 replicates per group. Error bars represent mean ± SD.

Figure S5 BM analysis for high- and low-precursor number groups

(A) RFP_{Conf}% distribution in BM HSPCs. Each dot represents one animal

(B) BM donor chimerism.

(C) BM nucleated cell numbers. N =15 mice for high precursor number group, n= 14 for low precursor number group. Unpaired t-test was performed.

(D) BM HSPC and MP frequency in donor CD45.2$^+$ population. Two-way ANOVA for paired samples was performed.

For (B) and (D), each solid dot represents one animal; each outlined dot represents the average value of a replicate containing multiple animals. Except for (C), recipient sample size = 7-14 mice per replicate, N =3 replicates per group. For all graphs, Error bars represent mean ± SD. ns, non-significant, * $p < 0.05$.

bioRxiv preprint doi: https://doi.org/10.1101/2024.04.02.587737; this version posted April 3, 2024. The copyright holder for this preprint (which was not certified by peer review) is the author/funder, who has granted bioRxiv a license to display the preprint in perpetuity. It is made available under aCC-BY 4.0 International license.

Figure S6 Analysis of FP induction in adult-induced animals

(A) Confetti labeling in LSK after 14days of tamoxifen treatment. N =5 animals. Error bars represent mean ± SD.
(B) CFP_{Conf}% in PB myeloid cells.
(C) CFP_{PB}% in PB myeloid cells.
(D) $RFP_{RFP+YFP}$% in PB myeloid cells.
(E) RFP_{PB}% and YFP_{PB}% in PB myeloid cells.
(F) CFP_{Conf}% in PB myeloid cells of fetal-induced animals
(G) BM HSPC frequencies. Error bars represent mean ± SD.

For (B-E) sample size = 14-22 mice per replicate; for (F), sample size = 4-11 mice per replicate; for (G) sample size = 8-10 mice per replicate. For (B-E) and (G), N =4 replicates. For (F), N =5 replicates.

Figure S7: Analysis related to 5-FU treatment and Confetti fetal induction

(A) PB myeloid cells frequencies before 5-FU treatment and ten days post-5-FU treatment. Dashed lines connect the values for the same replicate. Sample size = 6-8 mice per replicate, N =5 replicates for 5-FU. Paired t test was performed.
(B) The BM HSPC frequencies of UT and 5-FU treated groups ten days post-5-FU treatment. For UT, sample size = 8-10 mice per replicate, N =4 replicates; for 5-FU, sample size = 6-8 mice per replicate, N =5 replicates.
(C) Confetti% in PB in E11.5-labeled animals. Dashed lines connect values for the same replicate.
(D) The distribution of FPs in $Confetti^{+}$ population of B cells. Data showing average from all mice. N =24-34 for E14.5, N =7-24 for E11.5. Error bars represent mean ± SD.

For (A-C), each solid dot represents one animal; each outlined dot represents the average value of a replicate containing multiple animals. ns, non-significant; * $p < 0.05$, ** $p < 0.001$.

Figure S8 Analysis of FA mice at steady-state

(A) PB myeloid cell frequencies. Each dot represents one animal. Each outlined dot represents the average value of a replicate containing multiple animals.
(B) PB B cell frequencies. Each dot represents one animal. Each outlined dot represents the average value of a replicate containing multiple animals.
(C) Complete blood count analysis. Each dot represents one animal. N =13 for *Fancc*$^{+/+}$, N =16 for *Fancc*$^{-/-}$ mice. Unpaired t test was performed.
(D) BM HSPC frequencies. Each dot represents one animal. Each outlined dot represents the average value of a replicate containing multiple animals. One-way ANOVA was performed.
(E) BM nucleated cells counts. Each dot represents one animal. Unpaired t test was performed. N =12 for *Fancc*$^{+/+}$and *Fancc*$^{-/-}$ mice.

bioRxiv preprint doi: https://doi.org/10.1101/2024.04.02.587737; this version posted April 3, 2024. The copyright holder for this preprint (which was not certified by peer review) is the author/funder, who has granted bioRxiv a license to display the preprint in perpetuity. It is made available under aCC-BY 4.0 International license.

(F) $RFP_{RFP+YFP}$% in PB myeloid cells and B cells. Each dot represents one animal. Each outlined dot represents the average value of a replicate containing multiple animals.

For (A-B) and (F), Fancc$^{+/+}$, sample size = 11-17 mice per replicate, N =5 replicates; Fancc$^{+/-}$, sample size = 10-22 mice per replicate, N =7 replicates; Fancc$^{-/-}$, sample size = 9-17 mice per replicate, N =4 replicates. For (D), Fancc$^{+/+}$, sample size = 5-9 mice per replicate, N =5 replicates; Fancc$^{+/-}$, sample size = 6-12 mice per replicate, N =5 replicates; Fancc$^{-/-}$, sample size = 6-11 mice per replicate, N =4 replicates. Error bars represent mean ± SD. ns, non-significant; * $p < 0.05$.

Figure S9 Analysis of FA recipient mice

(A) Young *Fancc* donor BM HSPC frequency analysis. N = 5 for Fancc$^{+/+}$ or Fancc$^{+/-}$, N = 2 for Fancc$^{-/-}$.

(B) Donor chimerism in recipient BM of young *Fancc* donor. Each dot represents one animal. Each outlined dot represents the average value of a replicate containing multiple animals. Two-way ANOVA was performed. Recipient sample size = 7-10 mice per replicate, N =3 replicates per group.

(C) Number of PB B cell precursor in recipients of young FA donor.

(D) Number of PB T cell precursor in recipients of young FA donor.

(E) Number of BM HSPC precursor in recipients of young FA donor.

(F) Ageing *Fancc* donor BM HSPC frequency analysis. N = 3 for Fancc$^{+/+}$, N = 3 for Fancc$^{-/-}$.

(G) Number of PB myeloid cell precursor in recipients of aging FA donor.

(H) Number of PB T cell precursor in recipients of aging FA donor.

(I) Donor chimerism in recipient BM of ageing *Fancc* donor. Each dot represents one animal. Each outlined dot represents the average value of a replicate containing multiple animals. Two-way ANOVA was performed. Recipient sample size = 7-10 mice per replicate, N =3 replicates per group.

(J) Number of BM HSPC precursor in recipients of aging FA donor.

For (C-E), (G-H) and (J), N =3 replicates per group; permutation test was performed; dashed lines connect the average precursor numbers. Error bars represent mean ± SD. nd, not determined; ns, non-significant; * $p < 0.05$, ** $p < 0.001$. # represents lowest p value possible for permutation test.

bioRxiv preprint doi: https://doi.org/10.1101/2024.04.02.587737; this version posted April 3, 2024. The copyright holder for this preprint (which was not certified by peer review) is the author/funder, who has granted bioRxiv a license to display the preprint in perpetuity. It is made available under aCC-BY 4.0 International license.

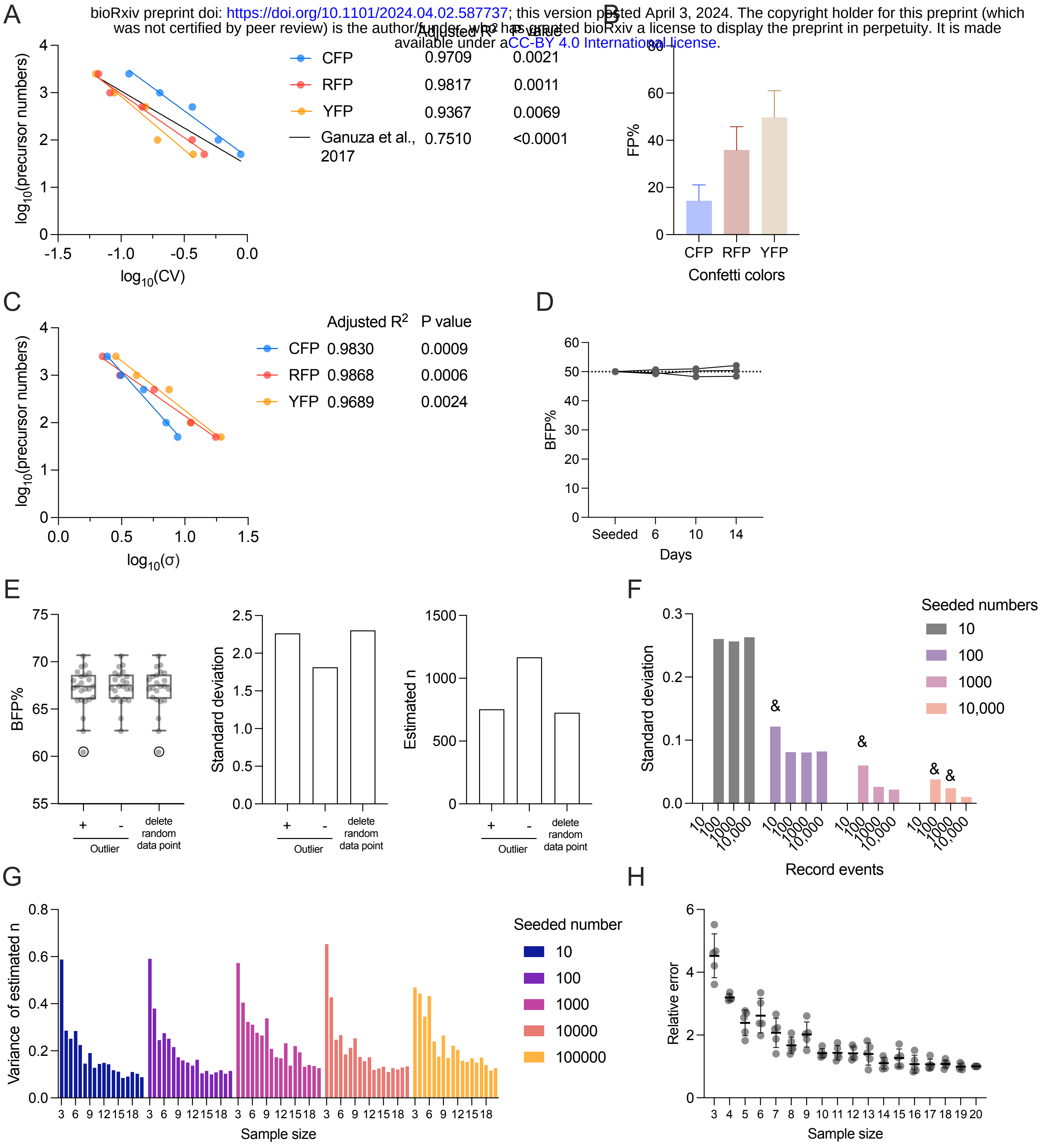

Figure S1

bioRxiv preprint doi: https://doi.org/10.1101/2024.04.02.587737; this version posted April 3, 2024. The copyright holder for this preprint (which was not certified by peer review) is the author/funder, who has granted bioRxiv a license to display the preprint in perpetuity. It is made available under aCC-BY 4.0 International license.

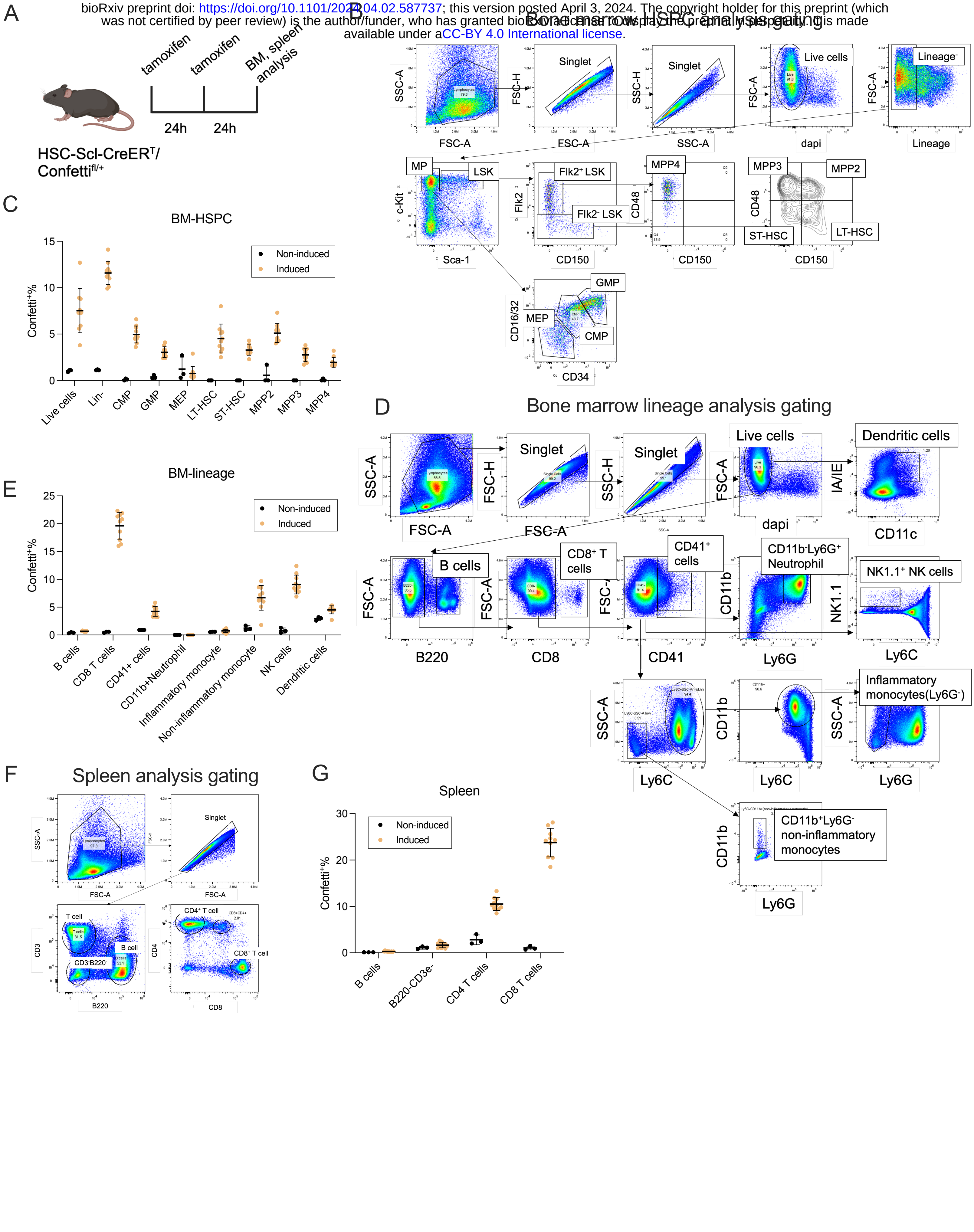

Figure S2

bioRxiv preprint doi: https://doi.org/10.1101/2024.04.02.587737; this version posted April 3, 2024. The copyright holder for this preprint (which was not certified by peer review) is the author/funder, who has granted bioRxiv a license to display the preprint in perpetuity. It is made available under aCC-BY 4.0 International license.

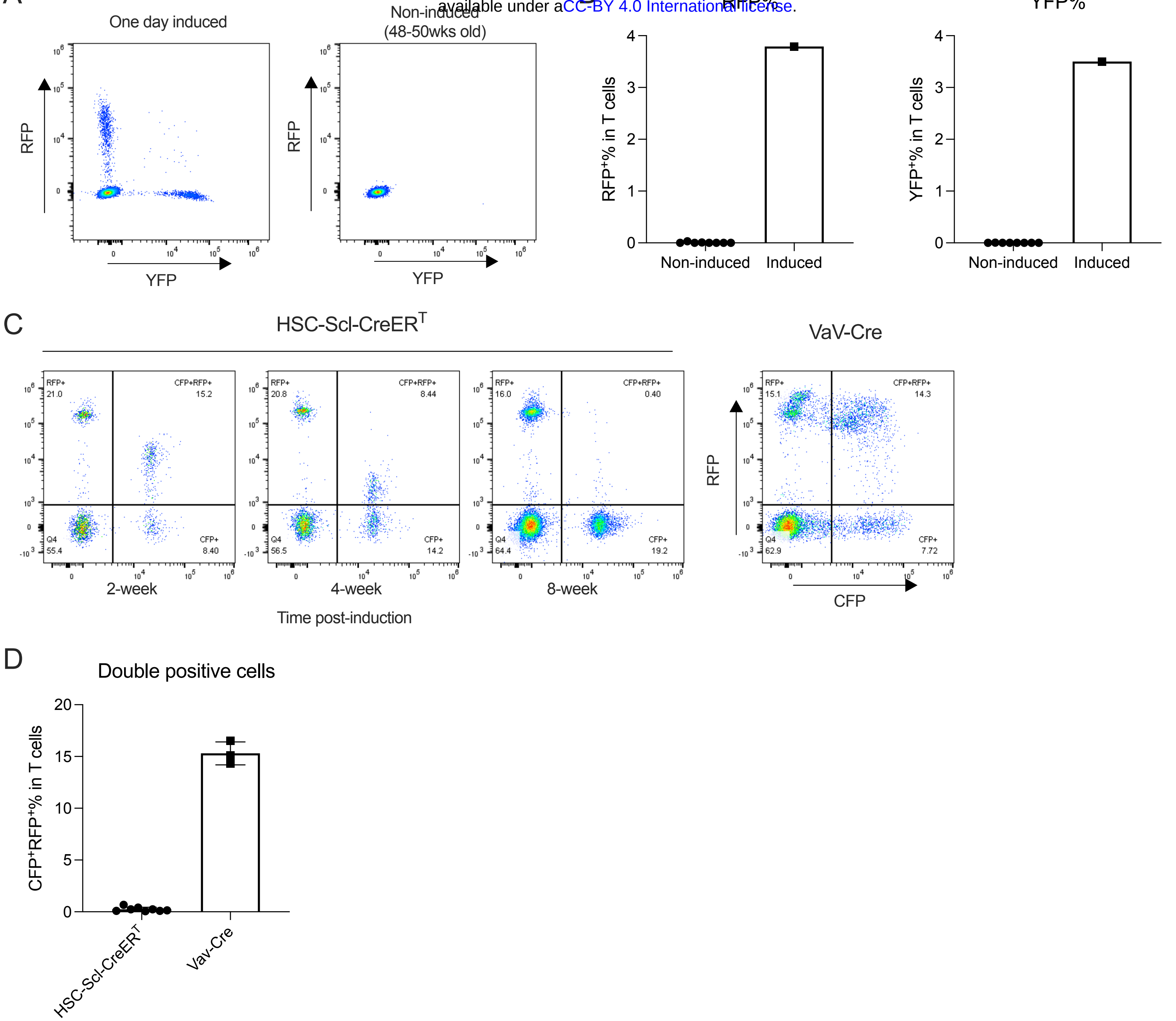

Figure S3

bioRxiv preprint doi: https://doi.org/10.1101/2024.04.02.587737; this version posted April 3, 2024. The copyright holder for this preprint (which was not certified by peer review) is the author/funder, who has granted bioRxiv a license to display the preprint in perpetuity. It is made available under aCC-BY 4.0 International license.

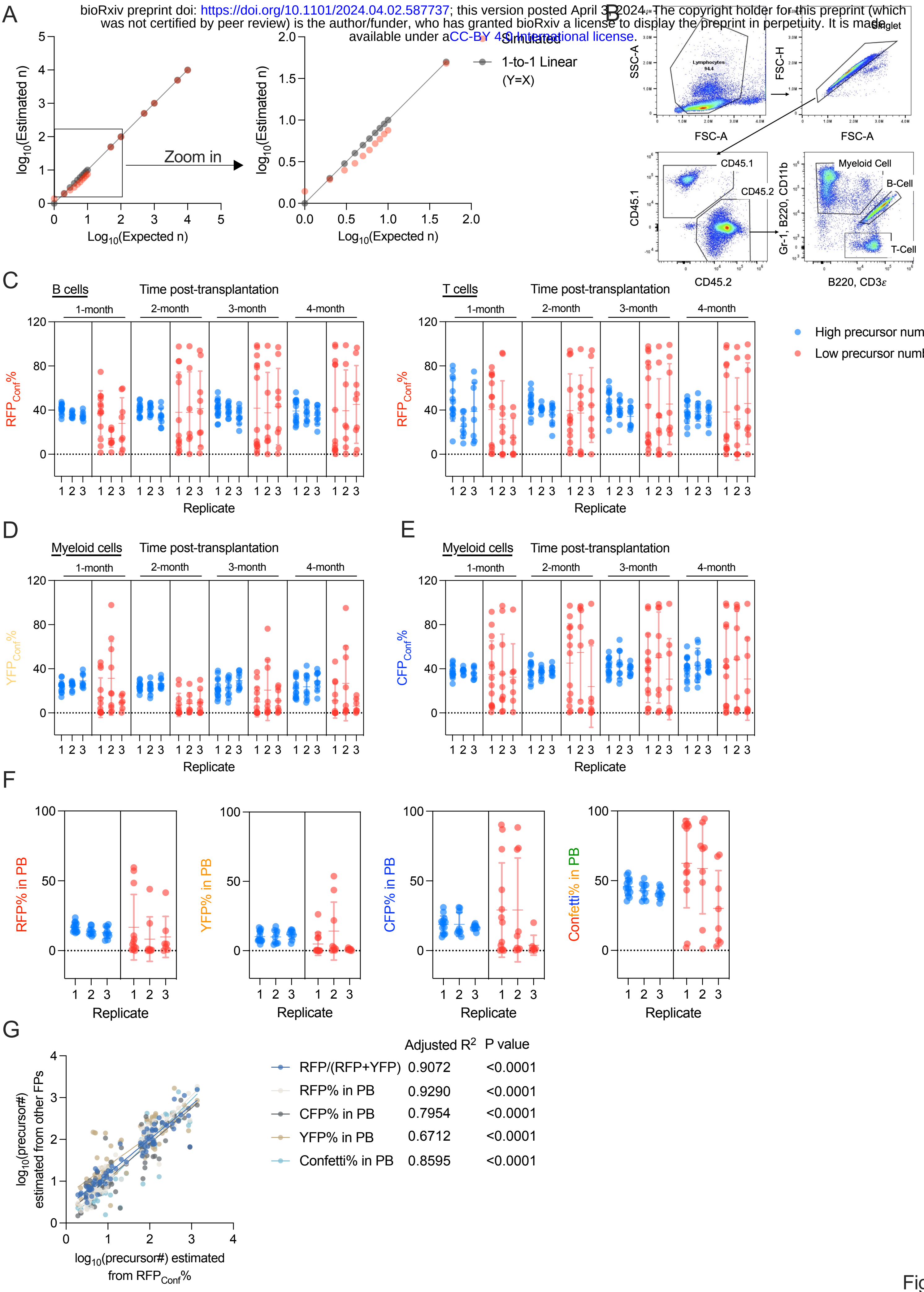

	Adjusted R^2	P value
RFP/(RFP+YFP)	0.9072	<0.0001
RFP% in PB	0.9290	<0.0001
CFP% in PB	0.7954	<0.0001
YFP% in PB	0.6712	<0.0001
Confetti% in PB	0.8595	<0.0001

Figure S4

bioRxiv preprint doi: https://doi.org/10.1101/2024.04.02.587737; this version posted April 3, 2024. The copyright holder for this preprint (which was not certified by peer review) is the author/funder, who has granted bioRxiv a license to display the preprint in perpetuity. It is made available under aCC-BY 4.0 International license.

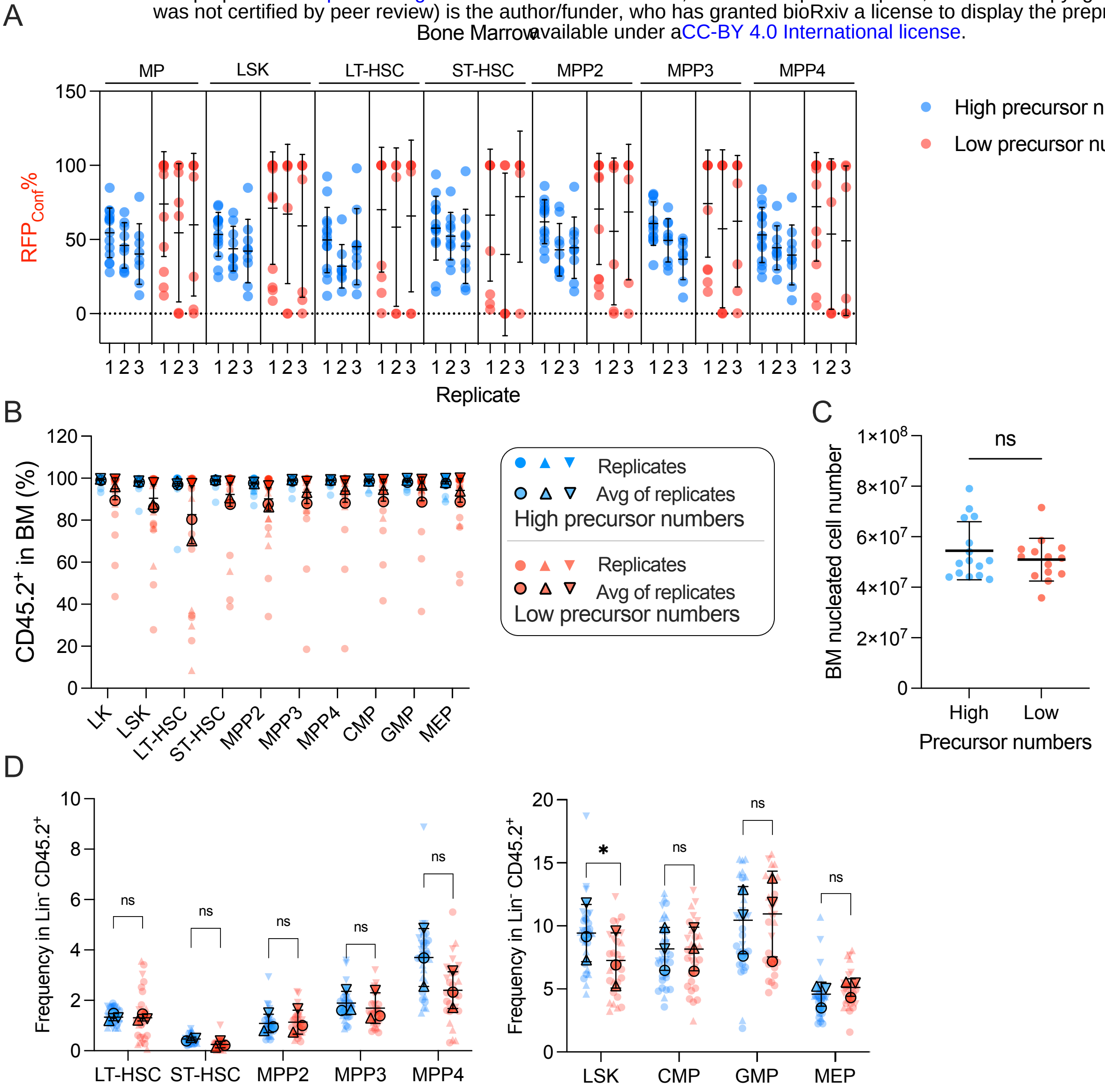

Figure S5

bioRxiv preprint doi: https://doi.org/10.1101/2024.04.02.587737; this version posted April 3, 2024. The copyright holder for this preprint (which was not certified by peer review) is the author/funder, who has granted bioRxiv a license to display the preprint in perpetuity. It is made available under aCC-BY 4.0 International license.

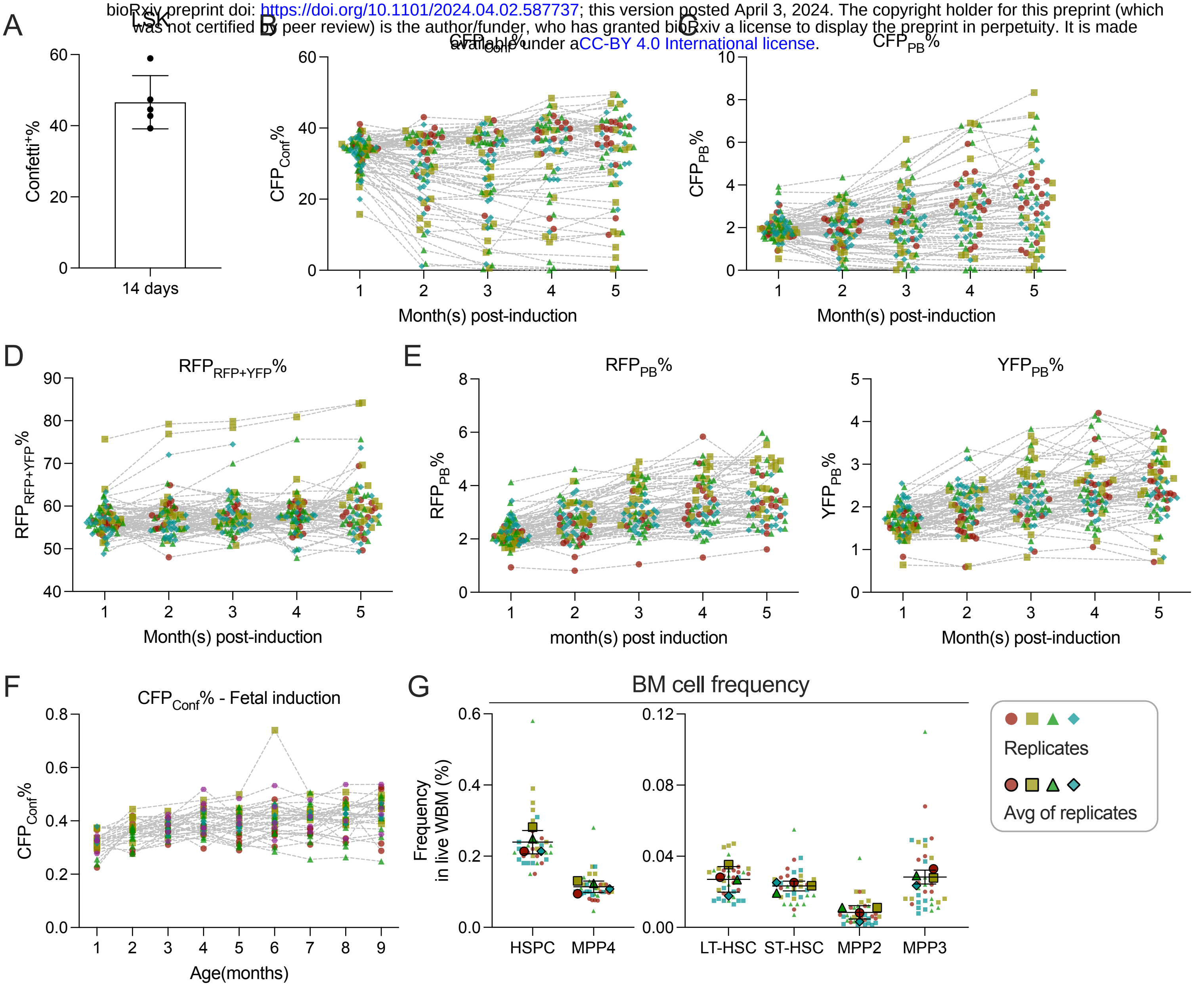

Figure S6

bioRxiv preprint doi: https://doi.org/10.1101/2024.04.02.587737; this version posted April 3, 2024. The copyright holder for this preprint (which was not certified by peer review) is the author/funder, who has granted bioRxiv a license to display the preprint in perpetuity. It is made available under aCC-BY 4.0 International license.

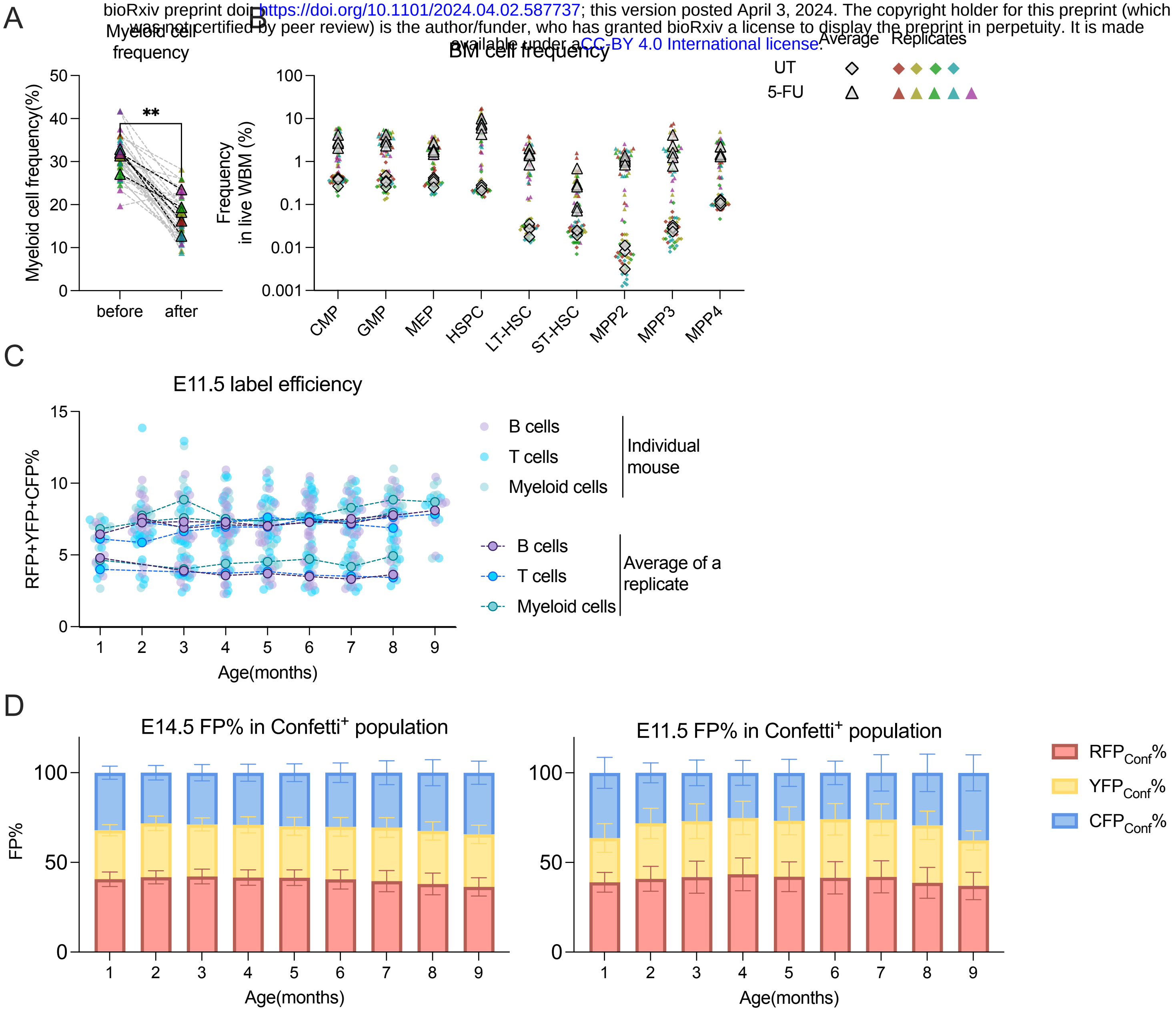

Figure S7

bioRxiv preprint doi: https://doi.org/10.1101/2024.04.02.587737; this version posted April 3, 2024. The copyright holder for this preprint (which was not certified by peer review) is the author/funder, who has granted bioRxiv a license to display the preprint in perpetuity. It is made available under aCC-BY 4.0 International license.

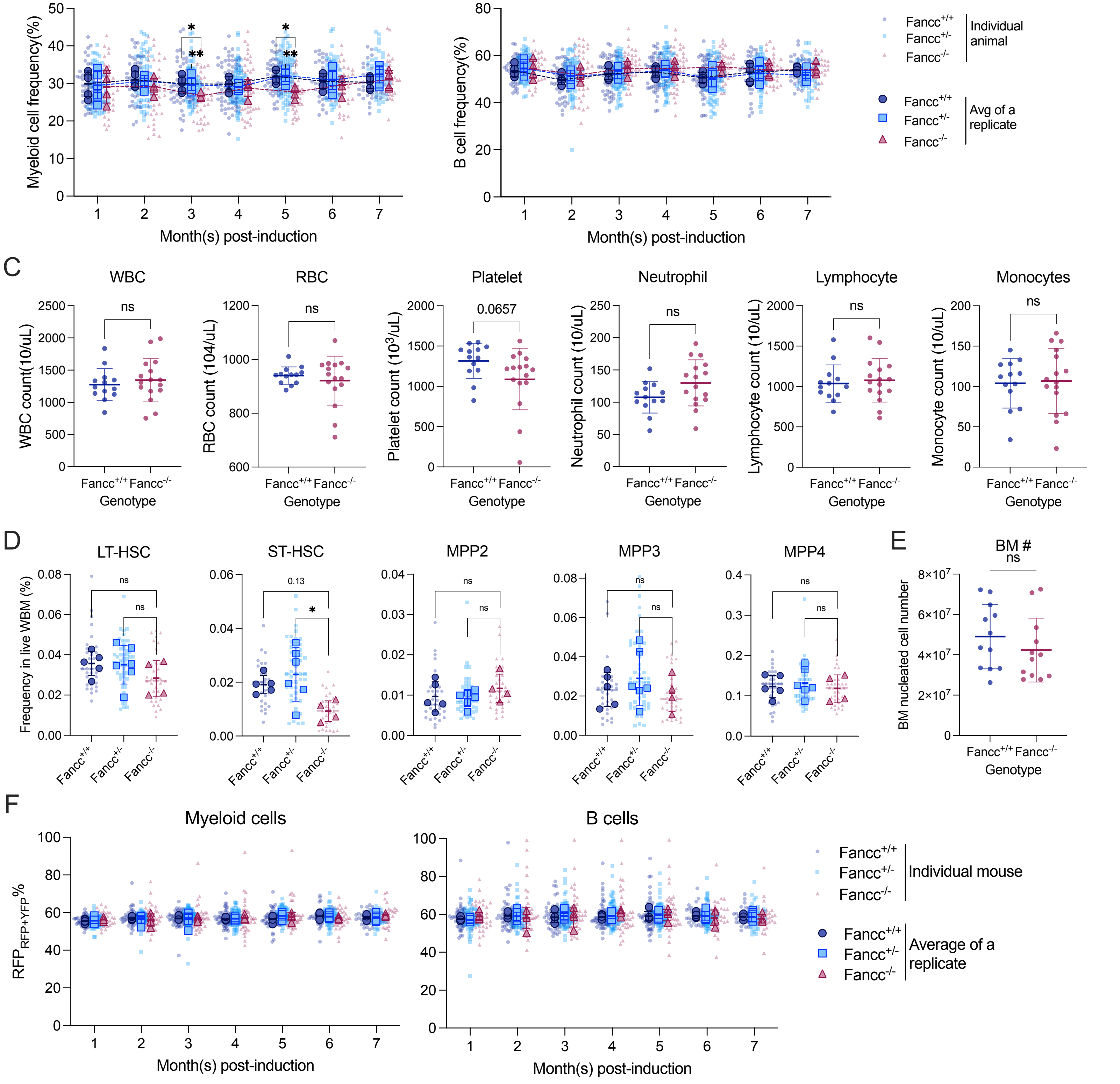

Figure S8

bioRxiv preprint doi: https://doi.org/10.1101/2024.04.02.587737; this version posted April 3, 2024. The copyright holder for this preprint (which was not certified by peer review) is the author/funder, who has granted bioRxiv a license to display the preprint in perpetuity. It is made available under aCC-BY 4.0 International license.

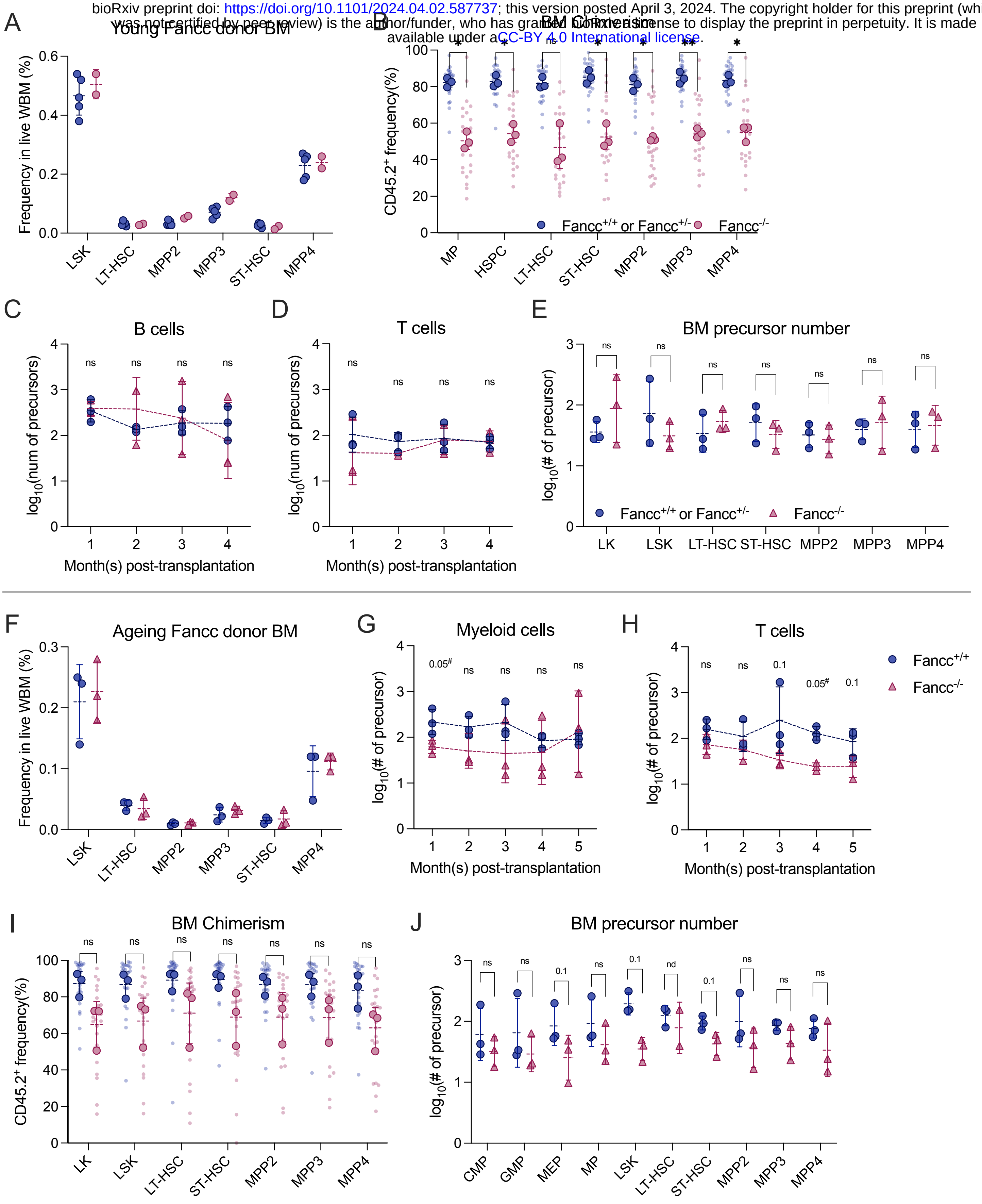

Figure S9
